# Draft Genome Sequence of the Lantibiotic-Producing Strain Streptococcus salivarius HS0302

**DOI:** 10.1128/MRA.01410-18

**Published:** 2019-01-03

**Authors:** Mengxin Geng, Peng Deng, Timothy Mire, Frank Austin, Leif Smith

**Affiliations:** a Department of Biology, Texas A&M University, College Station, Texas, USA; b Chinese Academy of Financial Inclusion, Renmin University of China, Beijing, China; c Department of Pathobiology and Population Medicine, College of Veterinary Medicine, Mississippi State University, Mississippi State, Mississippi, USA; Louisiana State University

## Abstract

Streptococcus salivarius is a prevalent commensal species of human oral mucosal surfaces. *S. salivarius* strain HS0302 produces the type AII lantibiotic salivaricin A2. Here, we report its draft genome sequence, revealing its potential to produce a variety of bacteriocins.

## ANNOUNCEMENT

Streptococcus salivarius is a predominant colonizer of oral mucosal surfaces in humans and does not cause disease in healthy individuals (1). Some commensal strains of *S. salivarius* have been used as probiotics for oral areas and the upper respiratory tract (2), including K12 (3) and M18 (4), due to their ability to produce a variety of peptide-based antibiotics which have a broad spectrum of activity against Gram-positive pathogens.

The type AII lantibiotic salivaricin A2 was discovered in 2006 and was shown to be active against Micrococcus luteus (5). Later, salivaricin A2 was demonstrated to be encoded at adjacent loci with salivaricin B on a 190-kb megaplasmid in a probiotic strain, *S. salivarius* K12 (6). However, the low production level of salivaricin A2 from the K12 strain limited the characterization of the lantibiotic. We previously reported the purification of salivaricin A2 from the HS0302 strain, which enabled the structure and bioactivity characterization of the lantibiotic (7). Here, we report its genome sequence, including the full sequence of the megaplasmid coding for two salivaricins and a draft chromosomal sequence.

Streptococcus salivarius HS0302 was cultured on a Todd-Hewitt yeast extract (THyex) agar plate (containing 30 g/liter Todd-Hewitt broth, 3 g/liter yeast extract, and 15 g/liter agar) or THyex broth. A single colony of the strain was inoculated into 10 ml THyex broth and was incubated overnight. Genomic DNA was isolated from the overnight culture using the Wizard genomic DNA purification kit (Promega) following the manufacturer’s instructions. Whole-genome sequencing was performed using the Illumina paired-end 150 platform (Novogene, Beijing, China). The library construction was done by Novogene. According to the report provided by Novogene, construction of the DNA libraries was performed using the processes of end repairing, the addition of A to tails, purification, and PCR amplification. Libraries were sequenced using an Illumina high-throughput sequencer with a paired-end sequencing strategy. Genome assembly was done using ABySS version 2.0.2, with default settings, and had 90× depth coverage. The draft *S. salivarius* HS0302 genome contains 32 contigs with an *N*_50_ value of 187,386 bp and has an approximate size of 2.33 Mb, with a G+C content of 39.38%. The sequence quality control (QC) method used was FastQC, with default settings (8). Gene annotation was performed using the NCBI Prokaryotic Genome Annotation Pipeline (9).

The *S. salivarius* HS0302 megaplasmid is 142,844 bp long (contig 18), indicated by the presence of the coding gene of a RepB family plasmid replication initiator protein. The megaplasmid encodes two salivaricins, including salivaricin A2 (119557 to 119712) and salivaricin 9 (105248 to 105418), which is unique among other reported *S. salivarius* strains. The whole sequence of the megaplasmid in *S. salivarius* HS0302 was compared with nucleotide BLAST to that of the *S. salivarius* K12 megaplasmid, which contains the salivaricin A2 and salivaricin B biosynthesis gene clusters. The comparison showed that the two megaplasmids share only 12% similarity. In addition to the two salivaricins, more than 10 putative bacteriocin biosynthesis gene clusters are identified from its chromosomal sequence through antiSMASH using default settings (10). It has been shown that one bacterium can contain genes for multiple bacteriocins; however, many of these genes are expressed only under certain circumstances, making the isolation process difficult (11). The draft genome sequence of *S. salivarius* HS0302 will be of interest for future studies aimed at identifying novel bacteriocins and for developing the strain into probiotics.

### Data availability.

This draft genome sequence has been deposited at DDBJ/ENA/GenBank under the accession no. NSIW00000000. The publicly available raw data are available under the SRA accession no. PRJNA400312.
